# Anti-integrin αvβ6 antibody in Takayasu arteritis patients with or without ulcerative colitis

**DOI:** 10.3389/fimmu.2024.1387516

**Published:** 2024-05-09

**Authors:** Yuki Ishikawa, Hiroyuki Yoshida, Hajime Yoshifuji, Koichiro Ohmura, Tomoki Origuchi, Tomonori Ishii, Tsuneyo Mimori, Akio Morinobu, Masahiro Shiokawa, Chikashi Terao

**Affiliations:** ^1^ Laboratory for Statistical and Translational Genetics, Center for Integrative Medical Sciences, RIKEN, Yokohama, Japan; ^2^ Department of Gastroenterology and Hepatology, Kyoto University Graduate School of Medicine, Kyoto, Japan; ^3^ Department of Gastroenterology, Kansai Electric Power Hospital, Osaka, Japan; ^4^ Department of Rheumatology and Clinical Immunology, Graduate School of Medicine, Kyoto University, Kyoto, Japan; ^5^ Department of Rheumatology, Kobe City Medical Center General Hospital, Kobe, Japan; ^6^ Department of Immunology and Rheumatology, Unit of Advanced Preventive Medical Sciences, Nagasaki University Graduate School of Biomedical Sciences, Nagasaki, Japan; ^7^ Clinical Research, Innovation and Education Center, Tohoku University Hospital, Sendai, Japan; ^8^ Department of Rheumatology, Ijinkai Takeada General Hospital, Kyoto, Japan; ^9^ Clinical Research Center, Shizuoka General Hospital, Shizuoka, Japan; ^10^ The Department of Applied Genetics, School of Pharmaceutical Sciences, University of Shizuoka, Shizuoka, Japan

**Keywords:** Takayasu’s arteritis, vasculitis, anti-integrin αvβ6 antibody, ulcerative colitis, HLA-B*52

## Abstract

**Background:**

It has been well documented that Takayasu arteritis (TAK) and ulcerative colitis (UC) coexist in the same patients. HLA-B*52 characterizes the co-occurrence, which is one of the common genetic features between these two diseases, indicating shared underlying pathologic mechanisms. Anti-integrin αvβ6 antibody (Ab) is present in sera of UC patients in a highly specific manner. We investigated if there were any associations between anti-integrin αvβ6 Ab and TAK, considering the risk HLA alleles.

**Methods:**

A total of 227 Japanese TAK patients were recruited in the current study and their serum samples were subjected to measurement of anti-integrin αvβ6 Ab by ELISA. The clinical information, including the co-occurrence of UC, was collected. The HLA allele carrier status was determined by Luminex or genotype imputation.

**Results:**

The information about the presence of UC was available for 165 patients, among which eight (4.84%) patients had UC. Anti-integrin αvβ6 antibody was identified in 7 out of 8 TAK subjects with UC (87.5%) while only 5 out of 157 (3.18%) TAK subjects without UC had the antibody (OR 121, p=7.46×10^-8^). A total of 99 out of 218 (45.4%) patients were HLA-B*52 carriers. There was no significant association between the presence of anti-integrin αvβ6 Ab and HLA-B*52 carrier status in those without UC (OR 2.01, 95% CI 0.33-12.4, p = 0.189).

**Conclusions:**

The prevalence of anti-integrin αvβ6 Ab was high in TAK patients with UC, but not in the absence of concomitant UC. The effect of HLA-B*52 on anti-integrin αvβ6 Ab production would be minimal.

## 1 Introduction

Takayasu arteritis (TAK) is a large-vessel vasculitis, that affects mainly aorta and its proximal branches potentially resulting in severe complications such as aortic regurgitation (1). In addition to environmental factors, genetic variations, especially single nucleotide polymorphisms (SNPs), have a significant role in the disease pathophysiology (2). Among genetic components, HLA-B*52 is the most significantly associated and hence an established risk locus of TAK susceptibility among different populations (3). Also, previous genome-wide association studies (GWASs) have identified significant disease-susceptible loci in the non-HLA region including *IL12B* (rs6871626) (4, 5), of which finding led to the usage of ustekinumab, an anti-IL12/23p40 monoclonal antibody, for TAK treatment through a successful pilot clinical trial result in Japan (6).

Ulcerative colitis (UC) is a chronic inflammatory bowel disease (IBD) and is characterized by the destruction of colonic epithelial cells leading to epithelial barrier defects. Immune dysregulation has been considered as a main pathologic feature of the disease, such as aberrant Th2 response and subsequent B-cell activation. Since specific autoantigens and the corresponding autoantibodies had not been identified, the disease diagnosis relied on clinical symptoms, colonoscopic findings, and histological features, which occasionally can be challenging (7). Recently, a breakthrough discovery has been made and anti-integrin αvβ6 antibody (Ab) has been identified to be present in sera of UC patients in a highly specific manner (8). Integrin avβ6 is a receptor for extracellular matrix proteins and is specifically expressed in epithelial cells, rendering integrated epithelial barrier functions. UC frequently co-exists in the same individuals with TAK (~ 6.4% in Japanese) (9), and importantly, TAK and UC share genetic components in a global manner including HLA-B*52 and rs6871626 in *IL-12B*, indicating the presence of shared underlying pathogenic mechanisms. We previously reported that HLA-B*52 characterizes the co-occurrence of TAK and UC with a strong effect size in an intra-case analysis of TAK (9).

In the present study, we investigated the presence of anti-integrin αvβ6 Ab in TAK patients with or without concomitant UC to address whether anti-integrin αvβ6 Ab could also play some roles in TAK pathology, which might be driven by shared genetic components between UC and TAK, especially HLA-B*52.

## 2 Materials and methods

### 2.1 Patients

A total of 227 Japanese TAK patients were recruited from the Kyoto University, Tohoku University, and Nagasaki University Hospital. TAK was diagnosed according to the criteria of the American College of Rheumatology (10, 11) or the guideline provided by the Japanese Circulation Society (12). The diagnosis of UC was based on the clinical, endoscopic, and histologic findings referring to the ECCO-ESGAR guideline (13) or the Japanese Society of Gastroenterology guideline (14, 15). Aortic regurgitation was assessed by echocardiography and/or angiography for its presence and severity. All subjects provided written informed consent. The study was approved by the Ethics Committee of Kyoto University Graduate School and Faculty of Medicine and the institutional review board of RIKEN Center for Integrative Medical Sciences.

### 2.2 Quantification of serum anti-integrin avβ6 antibody

Anti-integrin αvβ6 IgG Ab was measured using a commercially available enzyme-linked immunosorbent assay (ELISA) kit (5288; MBL, Japan) according to the manufacturer’s instruction. The cut-off value was based on the absorbance of negative control samples (a mean value plus 3 standard deviations) in the previous study, in which, plasma samples from UC patients, patients with non-UC, and healthy volunteers were tested for the presence of anti-integrin αvβ6 Ab (8).

### 2.3 Determination of HLA alleles

HLA alleles for HLA class I (-A, -B, and -C) and HLA class II (-DRB1, -DRB3, -DRB4, -DRB5, DQA1, -DQB1, and -DPB1) were determined by Luminex. For those who had not been genotyped by Luminex and whose DNA was available, DNA micro-array genotyping was conducted by Illumina Infinium Human Core Exome Array or Human Core Array in combination with Human Exome Array and genotype imputation was conducted with the use of SNP2HLA (v1.0, https://software.broadinstitute.org/mpg/snp2hla/).

### 2.4 Statistical analysis

Fisher’s exact test was applied to comparisons of categorical variables. Logistic regression model was applied to association tests using glm (fitting generalized linear models) function of R. All the statistical analyses were performed using R software (v4.0.3).

## 3 Results

**Figure 1:**
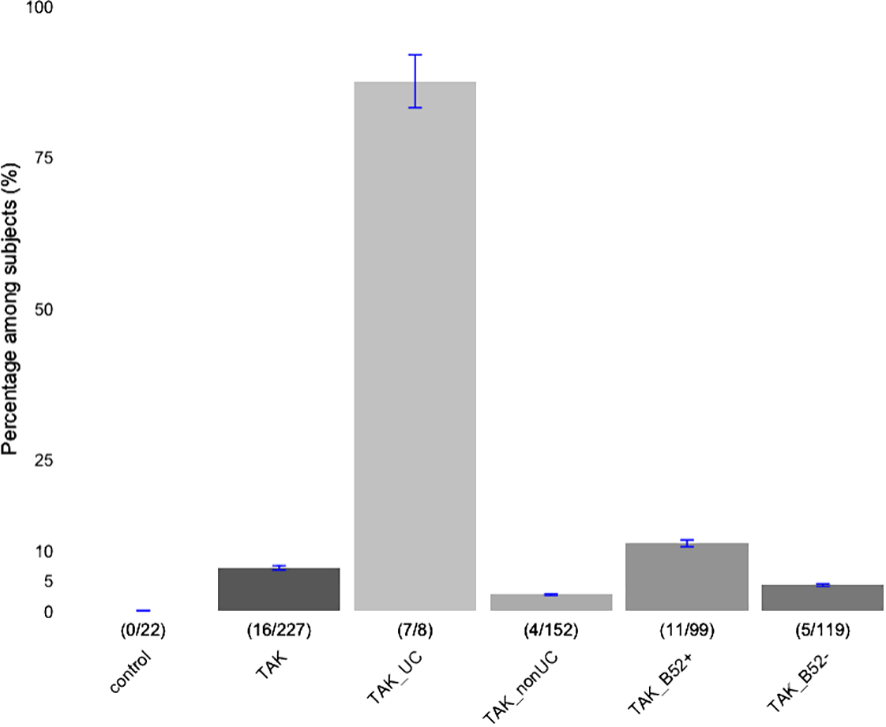
Percentages of subjects with anti-integrin αvβ6 antibody. Patients with Takayasu arteritis (TAK) were stratified based on the concomitant presence of ulcerative colitis (UC), or carrier status of HLA-B*52 (B52). A number of Subjects with anti-integrin αvβ6 Ab (numerator) in each subclass (denominator) are indicated inside the parenthesis. Those of healthy controls (control; reference 8) and entire TAK patients are presented in the first and second, respectively. The error bars indicate ±5% values.

A total of 227 TAK patients who had been tested for serum anti-integrin αvβ6 Ab during the study period were enrolled in the study. The percentages of subjects, entire TAK patients, and TAK patients stratified by the presence of UC or of HLA-B*52, who had anti-integrin αvβ6 Ab referring to the healthy control samples in the previous study (8) are presented in **Figure 1**. Among these, 16 (7.05%) were positive for the anti-integrin αvβ6 Ab (**Supplementary Table 1**). The information about the presence of UC was available for 12 out of 16 Ab-positive and 153 out of 211 Ab-negative patients. As expected, UC ratio, the fraction of subjects with UC, was significantly higher (0.583, 7 out of 12) among the subjects with anti-integrin αvβ6 Ab compared to those without anti-integrin αvβ6 Ab (0.0065, 1 out of 153) (**Supplementary Table 1**). When stratified by the presence of UC and the profile of anti-integrin αvβ6 Ab, 87.5% (7/8) of TAK with UC were positive for anti-integrin αvβ6 Ab, while only 3.18% (5/157) of non-UC TAK patients were positive for anti-integrin αvβ6 Ab (OR 121, 95% CI 13.3-5756.9, Fisher’s exact test p=2.99×10^-10^, **Supplementary Table 2**). While we confirmed the specificity of anti-integrin αvβ6 Ab to UC, we noted that a small fraction of patients also had anti-integrin αvβ6 Ab without co-occurrence of UC, as previously reported in patients with other diseases (8).

Since the strong association of HLA-B*52 with both TAK (4, 5) and UC (16, 17) has been well-established, especially in individuals that concomitantly have both etiologies (9), we investigated the carrier status of HLA-B*52 in our study samples. Nearly half of the subjects tested for the HLA genotypes were HLA-B*52 carriers (99 out of 218). The subjects with anti-integrin αvβ6 Ab (11 out 16) were more likely to be HLA-B*52 carriers than those without anti-integrin αvβ6 Ab (88 out of 202) (**Supplementary Table 1**; Fisher’s exact test OR 2.84, 95%CI 0.87-10.8, p=0.068). HLA-B*67 (18) and HLA-B*39 (19), both of which had also been reported for association with TAK, were not identified in our samples due to the limited sample size.

Then, we tested the association of anti-integrin αvβ6 Ab in the TAK subjects with or without concomitant UC taking account of the HLA-B*52 status among 165 TAK patients. Concordant with the previous finding, we confirmed the presence of anti-integrin αvβ6 Ab was highly specific to UC even among TAK patients; the presence of anti-integrin αvβ6 Ab was significantly associated with the presence of UC in TAK patients (OR 212.8, 95% CI 21.8-2074.2 p=3.94×10^-6^). Furthermore, the association was robust and independent of the carrying status of the well-established risk HLA alleles, HLA-B*52, and those previously reported and observed in our dataset, HLA-DRB1*04:05, and HLA-DRB1*15:02 (**Supplementary Table 3**). On the other hand, none of the risk HLA alleles above were independently associated with the presence of anti-integrin αvβ6 Ab (**Table 1**).

**Table 1:** Association between anti-integrin αvβ6 antibody and the known risk HLA alleles in TAK patients.

Model	Ab ~ UC + single HLA allele		Ab ~ UC + multiple HLA alleles	
	OR (95% CI)	P-value	OR (95% CI)	P-value
B*52	1.72 (0.31-9.48)	0.536	3.31 (0.30-36.2)	0.326
DRB1*04:05	7.32×10^-8^ (0-Inf)	0.993	7.56×10^-8^ (0-Inf)	0.993
DRB1*15:02	1.05 (0.17-6.48)	0.957	0.38(0.03-5.07)	0.467

Ab, antibody; UC, ulcerative colitis; 95% CI, 95% confidence interval; Inf, infinite number.

Since anti-integrin αvβ6 Ab was identified in the sera of 5 subjects, who had not presented UC, we further examined whether the presence of anti-integrin αvβ6 Ab in the non-UC subjects is driven by any of the above-mentioned TAK-risk HLA alleles, HLA-B*52, HLA-DRB1*04:05, and HLA-DRB1*15:02. We found that none of these HLA alleles were significantly associated with the presence of anti-integrin αvβ6 Ab in the TAK patients without UC (**Supplementary Table 4**). Together these results indicate that the presence of anti-integrin αvβ6 Ab in TAK patients is not driven by the known risk-HLA alleles.

Finally, we investigated a potential association of anti-integrin αvβ6 Ab with one of the serious complications of TAK, aortic regurgitation (AR) (**Supplementary Table 5**). Among subjects with anti-integrin αvβ6 Ab, neither TAK subjects with UC nor those without UC had developed AR, although the sample sizes were too small to conclude statistical significance.

## 4 Discussion

In the present study, we investigated the presence of anti-integrin αvβ6 Ab among Japanese TAK patients in the context of the coexistence of UC. As reported previously, anti-integrin αvβ6 Ab was identified in the subjects with UC in a highly specific manner (92.0% sensitivity and 94.8% specificity). In that study, only 1 or 2 of the subjects with non-UC diseases presented anti-integrin αvβ6 Ab (n=24-27) and none of the healthy controls (n=22) presented the antibody (8). On the other hand, though a small number of subjects in the current study, anti-integrin αvβ6 Ab was also identified in a substantial fraction of TAK subjects without UC (5 out of 182), which motivated us for further investigation of the underlying mechanisms considering the overlapping risks between TAK and UC (9). Then, we investigated the impact of HLA-B*52, a well-known risk allele both for TAK and UC, on the presence of anti-integrin αvβ6 Ab among our subjects, which revealed no significant association between anti-integrin αvβ6 Ab and HLA-B*52. All these results highlight the specificity of anti-integrin αvβ6 Ab in UC subjects regardless of the presence of its frequent comorbidity, TAK, or the carrier status of HLA-B*52.

In addition to anti-integrin αvβ6 Ab, various autoantibodies in TAK patients have been reported (20). Among them, anti-endothelial protein C receptor (EPCR) Ab is one of the autoantibodies present in TAK sera and was reported to be present in 34.6% of Japanese TAK patients (21). The presence of anti-EPCR Ab was significantly associated with the co-occurrence of UC in Japanese TAK patients (37.5%) (21), and 77.2% of primary UC patients derived from mixed populations of Japanese and the US had anti-EPCR Ab (22). The corresponding antigens of anti-integrin αvβ6 Ab and anti-EPCR Ab are both expressed on the extracellular domain of the intestinal epithelial plasma membrane (8, 22). The anti-integrin αvβ6 Ab competes with fibronectin for biding to αvβ6 leading to impaired epithelial integrity and antibody levels in UC were correlated with the degree of mucosal damage (8). On the other hand, EPCR plays a role in inhibiting cell adhesion molecules, chemokine production, and leukocyte adhesion, and its expression is reduced in IBD, leading to intestinal inflammation (23). Considering the distinct functional roles of the corresponding antigens, the generation of anti-integrin αvβ6 Ab and anti-EPCR Ab appears to be driven by distinct mechanisms including genetic risks. Further studies in UC subjects to address biological mechanisms underlying the production of anti-integrin αvβ6 Ab would be warranted.

Although GWASs for TAK (24, 25) have identified likely causal variants, their contribution to TAK pathology such as autoantibody production has yet to be well-clarified. Integrating GWAS and clinical information in future studies will enable the identification of links between genetic variations and clinical phenotypes, which will have a substantial impact on the management of TAK patients.

## Data availability statement

The individual genotype data and clinical information presented in this article are not readily available due to the ethical or privacy restriction policy of the IRBs in this study. Requests to access the datasets should be directed to the corresponding author.

## Ethics statement

The studies involving humans were approved by The Ethics Committee of Kyoto University Graduate School and Faculty of Medicine and The institutional review board of RIKEN Center for Integrative Medical Sciences. The studies were conducted in accordance with the local legislation and institutional requirements. The participants provided their written informed consent to participate in this study.

## Author contributions

YI: Writing – original draft, Writing – review & editing, Data curation, Formal analysis, Investigation, Software, Validation, Visualization. HiY: Resources, Writing – review & editing. HaY: Resources, Writing – review & editing. KO: Resources, Writing – review & editing. TO: Resources, Writing – review & editing. TI: Resources, Writing – review & editing. TM: Resources, Writing – review & editing. AM: Resources, Writing – review & editing. MS: Resources, Validation, Writing – review & editing. CT: Conceptualization, Data curation, Formal analysis, Funding acquisition, Investigation, Methodology, Project administration, Supervision, Validation, Writing – original draft, Writing – review & editing.
