# Supplementary material for: Anti-integrin αvβ6 antibody in Takayasu arteritis patients with or without ulcerative colitis
Source: Front Immunol. 2024 May 9;15:1387516. doi: 10.3389/fimmu.2024.1387516 (PMC11111853; doi:10.3389/fimmu.2024.1387516)
Supplement: Supplementary file 1 [file Table_1.docx]

Supplementary Material

**Supplementary Table 1. Demographic features of the patients with the profile of anti-integrin αvβ6 antibody.**

|  | All |  | Ab (+) |  | Ab (-) |
| --- | --- | --- | --- | --- | --- |
| Number of patients | 227 |  | 16 |  | 211 |
| Mean age of onset (years) + S.D | 31.1 + 15.9 |  | 22.1 + 13.2 |  | 31.8 + 15.9 |
| Female:Male ratio | 0.93:0.07 |  | 1.0:0 |  | 0.92:0.08 |
| Mean disease duration (years) + S.D | 19.3 + 15.8 |  | 24.4 + 13.1 |  | 18.8 + 15.9 |
| UC ratio# | 0.048 (8/165) |  | 0.583 (7/12) |  | 0.0065 (1/153) |
| AR ratio* | 0.412 (28/68) |  | 0 |  | 0.452 (28/62) |
| HLA-B*52 (B*52, B*52:01) | 0.454 (99/218) |  | 0.688 (11/16) |  | 0.436 (88/202) |

#UC ration, a fraction of subjects having ulcerative colitis; *AR ratio, a fraction of subjects having aortic regurgitation

**Supplementary Table 2. Stratification of TAK patients by the presence of UC, the anti-integrin αvβ6 Ab profiles, and carrier status of risk HLA alleles.**

|  | UC (8) | | Non-UC (157) | |
| --- | --- | --- | --- | --- |
|  | Ab (+) N=7 | Ab (-) N=1 | Ab (+) N=5 | Ab (-) N=152 |
| HLA-B*52 (+) | 6 (85.7%) | 1 (100%) | 3 (60.0%) | 65 (42.8%) |
| HLA-B*52 (-) | 1 (14.3%) | 0 | 2 (40.0%) | 87 (57.2%) |
| HLA-DRB1*04:05 (+) | 0 (0%) | 0 | 0 | 37 (24.3%) |
| HLA-DRB1*04:05 (-) | 7 (100%) | 1 (100%) | 5 (100%) | 115 (75.7%) |
| HLA-DRB1*15:02 (+) | 7 (100%) | 1 (100%) | 2 (40%) | 59 (38.8%) |
| HLA-DRB1*15:02 (-) | 0 (0%) | 0 | 3 (60%) | 93 (61.2%) |

**Supplementary Table 3. Association between anti-integrin αvβ6 antibody and UC conditioned on the known risk HLA alleles.**

| Allele(s) conditoned on | OR (95% CI) of UC | P-value |
| --- | --- | --- |
| *B*52* | 175.6 (17.1-1799.9) | 1.35×10^-5^ |
| *DRB1*04:05* | 161.0 (16.5-1571.9) | 1.24×10^-5^ |
| *DRB1*15:02* | 206.5 (16.5-2579.5) | 3.51×10^-5^ |
| Above three alleles | 192.2 (14.1-2623.7) | 8.04×10^-5^ |

TAK, Takayasu arteritis; UC, ulcerative colitis; 95% CI, 95% confidence interval

**Supplementary Table 4. Association between anti-integrin αvβ6 antibody and the known risk HLA alleles in TAK patients without UC.**

| Model | Ab ~ single HLA allele | | Ab ~ multiple HLA alleles | |
| --- | --- | --- | --- | --- |
|  | OR (95% CI) | P-value | OR (95% CI) | P-value |
| *B*52* | 2.01 (0.33-12.4) | 0.452 | 5.41 (0.43-67.3) | 0.189 |
| *DRB1*04:05* | 7.32×10^-8^ (0-Inf) | 0.993 | 2.83×10^-8^ (0-Inf) | 0.995 |
| *DRB1*15:02* | 1.05 (0.17-6.48) | 0.957 | 0.27 (0.02-3.37) | 0.308 |

Ab, antibody; UC, ulcerative colitis; 95% CI, 95% confidence interval; Inf, infinite number

**Supplementary Table 5. Stratification of TAK patients by the presence of UC, the anti-integrin αvβ6 Ab profiles, and the presence of aortic regurgitation.**

|  | **UC (4)** | | **Non-UC (90)** | |
| --- | --- | --- | --- | --- |
|  | **Ab (+)** | **Ab (-)** | **Ab (+)** | **Ab (-)** |
|  | 3 | 0 | 3 | 62 |
| **AR (+)** | 0 | 0 | 0 | 28 |
| **AR (-)** | 3 | 0 | 3 | 34 |

UC, ulcerative colitis; AR, aortic regurgitation
